# *Bacillus*‐based probiotic cleansers reduce the formation of dry biofilms on common hospital surfaces

**DOI:** 10.1002/mbo3.1391

**Published:** 2023-11-22

**Authors:** Richard Wormald, Paul N. Humphreys, Christopher J. Charles, Simon P. Rout

**Affiliations:** ^1^ Department of Biological and Geographical Sciences University of Huddersfield Huddersfield UK; ^2^ Genesis Biosciences Cardiff UK

**Keywords:** *Bacillus*, biofilm, CLSM‐FISH, probiotic, surface

## Abstract

In the absence of liquid suspension, dry biofilms can form upon hard surfaces within a hospital environment, representing a healthcare‐associated infection risk. Probiotic cleansers using generally recognized as safe organisms, such as those of the *Bacillus* genus, represent a potential strategy for the reduction of dry biofilm bioburden. The mechanisms of action and efficacy of these cleaners are, however, poorly understood. To address this, a preventative dry biofilm assay was developed using steel, melamine, and ceramic surfaces to assess the ability of a commercially available *Bacillus* spp. based probiotic cleanser to reduce the surface bioburden of *Escherichia coli* and *Staphylococcus aureus*. Via this assay, phosphate‐buffered saline controls were able to generate dry biofilms within 7 days of incubation, with the application of the probiotic cleanser able to prevent >97.7% of dry biofilm formation across both pathogen analogs and surface types. Further to this, surfaces treated with the probiotic mixture alone also showed a reduction in dry biofilm across both pathogen and surface types. Confocal laser scanning microscopy imaging indicated that the probiotic bacteria were able to germinate and colonize surfaces, likely forming a protective layer upon these hard surfaces.

## Graphical abstract

We developed a test method for assessing the prevention of the adhesion of organisms to surfaces that could generate dry biofilm material. The study used a probiotic blend of *Bacillus* and a formulated product to assess their efficacy and visualize the presence of pathogen analogs on the surface of three material types.

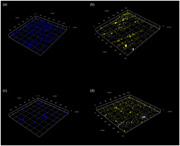

## 1 INTRODUCTION

Within the clinic environment, dry biofilms have been described as any biofilm that is capable of forming upon a dry surface, while wet biofilms are those more closely associated with medical devices such as catheters (Ledwoch et al., 2018). Recent studies have indicated that organisms, including microbial pathogens, can survive on a multitude of surfaces, likely as dry biofilms, ranging from everyday items such as keyboards and folders (Ledwoch et al., 2018) to those of clinical relevance such as hard surfaces within the hospital environment (Caselli et al., 2016). The control of dry biofilms within a hospital setting is therefore important to manage surface pathogens and associated antimicrobial resistance gene loads. The dry biofilm mode of existence is thought to provide a protective niche that leads to reservoirs of clinically relevant pathogens. These protective niches, much like those found in wet biofilm, provide a resistance mechanism against environmental stressors and xenobiotic compounds. Conventional disinfection methods, such as the use of hypochlorite, are less effective against dry biofilms than against planktonic cells of *Staphylococcus aureus* (Almatroudi et al., 2016). Additional studies have also suggested the limitations of quaternary ammonium compounds and thermal treatment against dry biofilms (Almatroudi et al., 2018; Lineback et al., 2018). Dry biofilms, therefore, represent a viable target for the reduction of hospital‐acquired infections (HAIs) and the increased associated economic burden through surface pathogen control. Increasing resistance mechanisms to chemical treatments represent an increasing cost to healthcare services, with estimated treatment costs from healthcare‐associated infections reaching £1bn in the United Kingdom (Ebrahimi et al., 2017; Mackley, 2018).

In the search for alternative solutions to combat HAI and its causative agents, probiotic treatments of surfaces have been suggested by a number of studies (De Cesare et al., 2019; D'accolti et al., 2018, 2019). Here, the approach aims to use a nonpathogenic microorganism to colonize hard surfaces and effectively outcompete clinically relevant pathogens. The technologies available generally combine hard surface cleaning product formulations such as surfactants, chemical preservatives, and stabilizers to offer both an immediate biocidal challenge via the chemical formulation and a longer‐lasting impact via the biological agent. One of the major biological agents used for this approach is various species of the *Bacillus* genus. These are chosen due to their ability to be grown with relative ease at the industrial scale, coupled with their ability to form highly stable spores. These spores offer a prolonged product shelf life and increased flexibility with cleaning product formulations compared to preserved planktonic cells. Further to this, the approach of using *Bacillus* spp. aims to exploit the diverse antimicrobial secondary metabolite capabilities found within the different members of the genus to deliver a more natural and precise mode of pathogen control. Recently, this concept has been demonstrated in the clinical setting via multicenter hospital trials in Italy (Caselli et al., 2018, 2019). Within these studies, the use of *Bacillus* spp. based probiotic cleansers indicated a reduction in HAI from 4.8% to 2.3% over 18 months (Caselli et al., 2018), demonstrating the suitability of this approach for the prevention of pathogen accumulation upon hard surfaces.

Observations from multicenter studies (Caselli et al., 2018, 2019) offer a good initial insight as to the efficacy of using probiotic‐based cleansers toward the control of HAI; however, the underpinning microbiology as to the exact mechanisms of action, especially in relation to the dry biofilm mode of life, is still somewhat lacking, especially regarding the ability of *Bacillus* spp. spores to germinate and colonize hard surfaces. A reproducible model system is therefore needed to both improve scientific understanding and provide a tool to better engineer probiotic technologies toward hard surface pathogen control. There have been methods proposed for both the generation of dry biofilms on surfaces using a variety of static (Adator et al., 2018) and mixed reactor approaches (Almatroudi et al., 2015) with varying levels of exposure to liquid media requiring up to 12 days to generate and using specialist equipment such as a CDC biofilm reactor (Almatroudi et al., 2015, 2016, 2018). However, the current methodologies are typically considered for a reactive approach to testing such that a biofilm is developed on the surface before being treated through chemical interventions. Within the present study, we have developed a simple and reproducible model for assessing the ability of probiotic treatment of surfaces in a preventative study design for dry surface biofilms.

## 2 MATERIALS AND METHODS

### 2.1 Probiotic cleanser

The probiotic cleanser used was a commercially available general all‐purpose cleaner provided by Genesis Biosciences. The product consists of a blend of *B. amyloliquefaciens, B. licheniformis, B. subtilis, B. pumilus*, and *B. megaterium* spores at a combined concentration of 6.7 × 10^7^ CFU/mL suspended in a liquid cleaning formulation that contained nonionic surfactants, benzothiazolinone (BIT) preservatives, and organic based chelation agents. A cleanser formulation‐free *Bacillus* blend was prepared in the same manner, with the liquid cleaning formulation replaced with phosphate‐buffered saline (PBS).

### 2.2 Dry biofilm formation

Dry biofilms were formed on the test surfaces using the modified method of Adator et al. (2018). Briefly, test microorganisms (*Staphylococcus aureus* NCIMB 9518 or *Escherichia coli* NCIMB 8879) were prepared to a concentration of 1.5–5.0 × 10^8^ CFU/mL from 48‐h stock plates on tryptone soya agar (TSA; Neogen) in maximum recovery diluent (MRD; Neogen) and 50 µL deposited onto the test surface. Three test surfaces were selected, namely, stainless steel (3.1 cm^2^; SYSPAL), melamine (4.0 cm^2^; Wickes), and ceramic (4.0 cm^2^; Wickes), that were sterilized through autoclaving before use. The treated surfaces were then left at room temperature for 7 days in a sterile environment at room temperature and humidity. To assess the viability of the dry biofilm, surviving organisms were enumerated following an initial rinse of the test piece with 1 mL of sterile PBS to remove loosely attached cells; test pieces were face down into a sterile 5 cm Ø pot containing 10 mL of Dey–Engley neutralizing broth (Neogen) and 5 g glass beads (3 mm Ø). Following orbital shaking at 120 rpm for 30 min at room temperature, a range of dilutions of the resulting mixture was prepared in PBS and plated out onto TSA. Surviving organisms were then determined following incubation at 37°C. A set of test pieces was also treated with uninoculated MRD as a negative control.

### 2.3 Spore status following application

To understand the extent to which germination was occurring through the applied *Bacillus* spores, the spore blend or formulated product (100 µL) was dispensed on each test surface type (*n* = 6) and left for 1 h at room temperature. After this time, the organisms were recovered from the surface using the method described in 2.2; following orbital shaking, three of the replicates were enumerated as per 2.2 to give a total number of cells, while the remaining three replicates were heat shocked at 80°C for 20 min before TSA enumeration to give a spore count, with the difference between these two being the estimate of vegetative cells.

### 2.4 Antidry biofilm assay

To determine the extent of dry biofilm prevention by the probiotic blend of *Bacillus*, surfaces were pretreated with the following: (1) the probiotic cleanser (*Bacillus* + formulation), (2) the *Bacillus* blend prepared in PBS, and (3) PBS as a control. In treatments containing *Bacillus*, a concentration of 1.0–3.0 × 10^7^ CFU/mL was confirmed by dilution of the product in PBS and plating out onto TSA, followed by incubation at 37°C for 24 h. Each test surface was treated in triplicate with 100 µL cleanser, *Bacillus* blend, or control and left at room temperature until visibly dry, ∼1 h. The treated surfaces were then challenged with 50 µL of the 1.5–5.0 ×10^8^ CFU/mL suspension of microorganisms and incubated at room temperature for 7 days. The surviving organisms were then recovered as per the section above. To differentiate between *Bacillus* spp. and surviving challenge organism, *S. aureus* was recovered on Baird Parker Agar (Neogen) and *E. coli* was recovered on MacConkey agar (Neogen) supplemented with 0.5% sodium deoxycholate. Before testing, both media types were validated to ensure that *Bacillus* spp. were inhibited.

### 2.5 CLSM imaging

Initially, surfaces (steel, melamine, and ceramic) were inoculated with 100 µL of *Bacillus* blend (1.5 × 10^7^ CFU/mL) or PBS, then left to air dry for 1 h (or until visibly dry). Once dried, 50 µL of the 1.5–5.0 × 10^8^ CFU/mL microorganism was added to the surface and then left at room temperature for 7 days. After this, surfaces were rinsed with 1 mL PBS to remove transient organisms. Fluorescence in situ hybridization (FISH) of the inoculated surfaces was performed using previously described methods (Ainsworth et al., 2006). The buffer used for the hybridization was composed of 0.9 M NaCl, 0.01% sodium dodecyl sulfate (SDS), 0.01 M Tris‐HCl (pH 7.2), and 35% formamide, with all probes (Table 1) used at a final concentration of 5 ng/µL. The hybridization was conducted at 46°C for 1.5 h, followed by a 10‐min wash in a buffer (0.08 M NaCl, 0.01% SDS, and 0.01 M Tris‐HCl (pH 7.2)) at 46°C. FISH confocal laser scanning microscopy (FISH‐CLSM) of samples was performed at the Bioimaging Facility at the University of Huddersfield, UK, using a Zeiss LSM880 inverted confocal microscope, and images were processed using Zen 2.1 software (Zeiss Microscopy).

**Table 1:** Fluorescent probes were used in the study.

Probe	Target	Sequence (5′–>3′)	Fluorochrome	Reference
BAC1	*Bacillus* spp.	ATG ATG GTG ACG GCG TTG GGG CAG GAA GA	Cy3	Kudoh and Ikeuchi (1985)
ECO1482	*Escherichia coli*	TAC GAC TTC ACC CCA GTC	Cy5	Tang et al. (2005)
Sau66	*Staphylococcus aureus*	AAG CTT CTC GTC CGT TCG	Cy5	Wang (2010)

## 3 RESULTS

### 3.1 Dry biofilm development on surfaces

**Figure 1:**
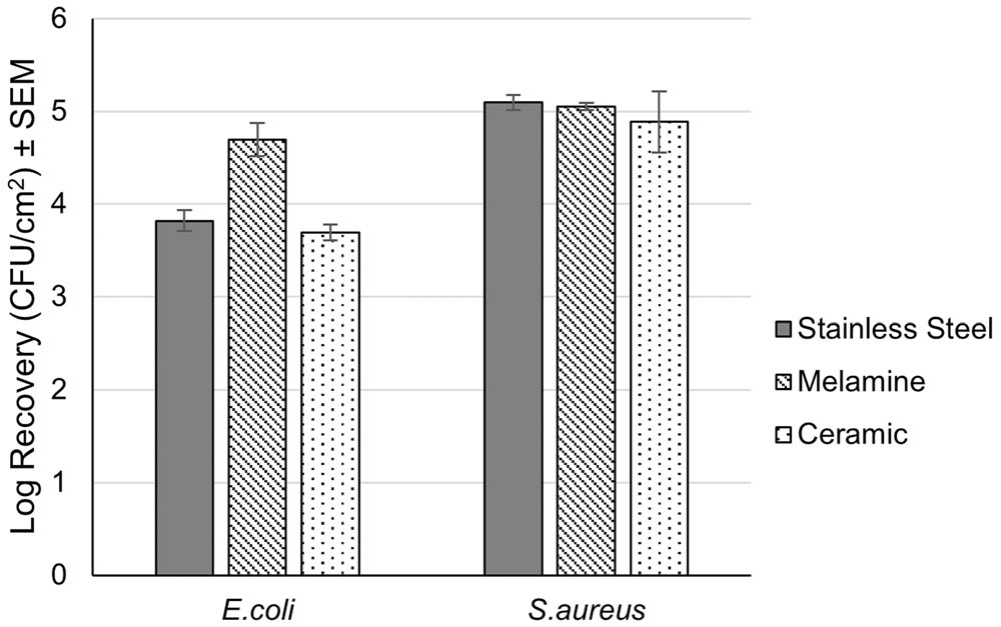
Log recovery of surviving organisms across the three surface types when inoculated with *Escherichia coli* and *Staphylococcus aureus* after 7 days of incubation at room temperature and RH, *n* = 3. CFU, colony‐forming unit; RH, relative humidity.

The dry biofilm model system was able to generate biofilms on the surface of all 3 coupon types (stainless steel, ceramic, and melamine) after 7 days of incubation at room temperature (Figure 1). Surfaces inoculated with *E. coli* showed the greatest degree of variation by surface, with 3.7–3.8 Log organisms recovered per cm^2^ on stainless steel and ceramic, respectively, while 4.7 Log organisms were recovered from melamine. Recovery of *S. aureus* was more consistent, ranging between 4.9 and 5.1 Log organisms across the three test surfaces.

### 3.2 Status of *Bacillus* spp. on inoculated surfaces

**Figure 2:**
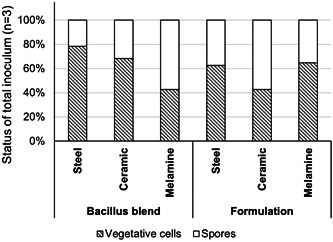
Status of *Bacillus* spp. recovered from steel, ceramic, and melamine surfaces indicated the presence of both vegetative cells and spores present on all surface types.

Recovery of the *Bacillus* spp. inoculated on each of the surfaces to determine the number of vegetative cells and spores (Figure 2) indicated that both vegetative cells and spores were present following an hour of incubation at room temperature. On surfaces that were inoculated solely with the blend of *Bacillus* strains, vegetative cells on the surfaces ranged from 43% to 78%, being highest on the steel surfaces and least on melamine. The range of vegetative cells on the surface was narrower upon surfaces treated with the formulated *Bacillus* blend, ranging from 43% to 65%, with ceramic having the lowest proportion of vegetative cells. The total recovery of *Bacillus* from each test surface can be seen in Table A1.

### 3.3 Anti‐dry biofilm properties of the *Bacillus* blend

**Figure 3:**
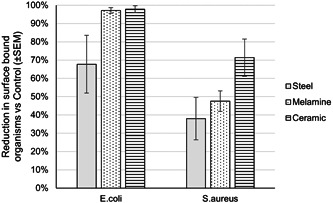
The *Bacillus* blend, with no additional cleaning chemistry, was capable of decreasing the microbial load on all three test surfaces when challenged with both *Escherichia coli* and *Staphylococcus aureus* when compared to the phosphate‐buffered saline control.

The application of the *Bacillus* blend, without additional cleaning chemistry, was able to decrease the surface colonization of both *E. coli* and *S. aureus* when compared to the PBS control, as determined via enumeration techniques (Figure 3). In comparison to the PBS control, the presence of *E. coli* on both melamine and ceramic was reduced by >97%, while a reduction of 67.7% ± 15.7% was observed on the steel surfaces. For *S. aureus*, reductions in microbial load after *Bacillus* blend treatment were much lower than that of *E. coli*, with ceramic yielding a reduction of 71.3%.

**Figure 4:**
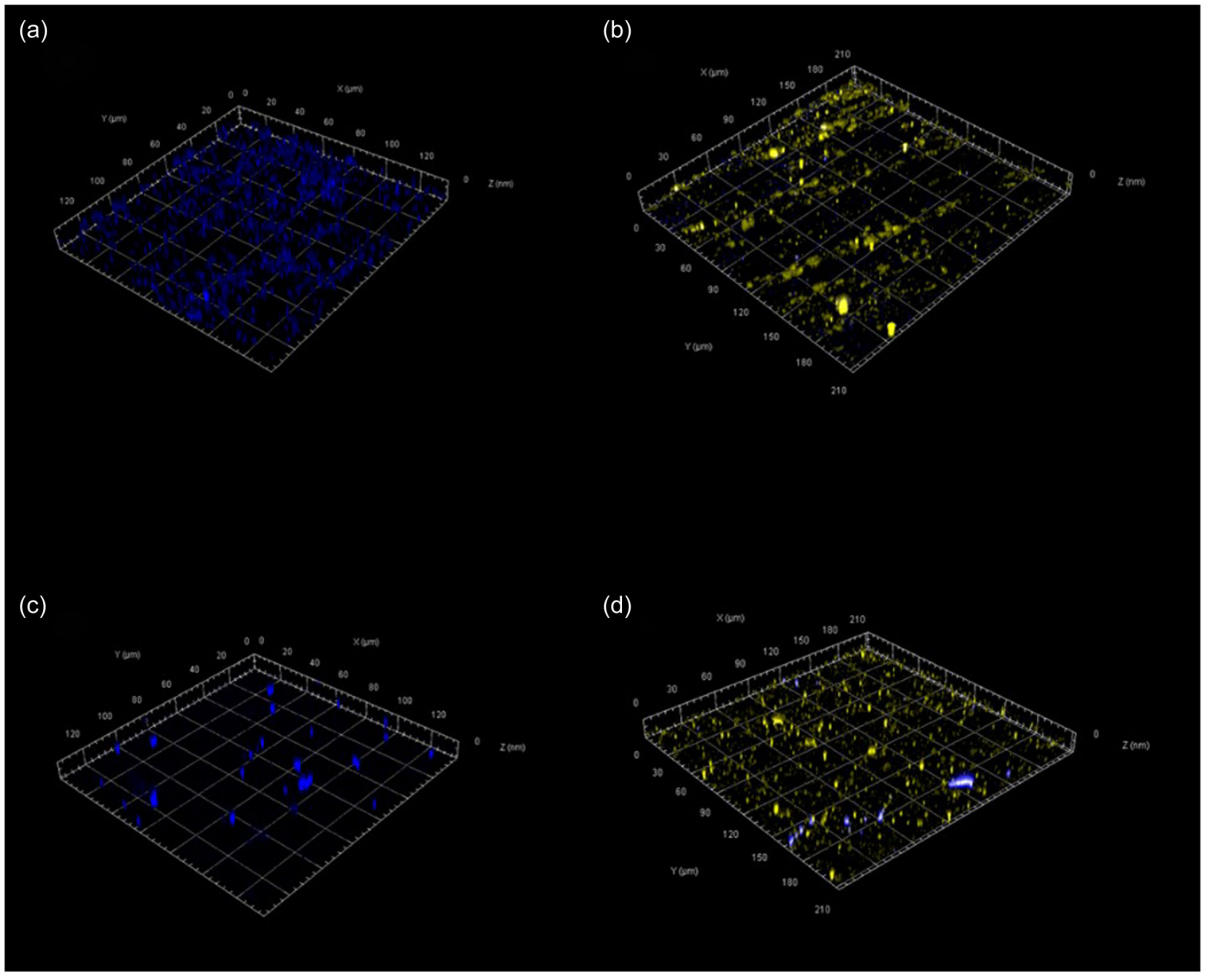
Example confocal laser scanning microscopy images showing dry biofilm formation of *Escherichia coli* (a) and *Staphylococcus aureus* (c) on stainless steel surfaces fluorescing blue. Stainless steel surfaces pretreated with the *Bacillus* blend before the addition of *E. coli* (b) and *S. aureus* (d) are shown with *Bacillus* fluorescing yellow.

**Figure A1:**
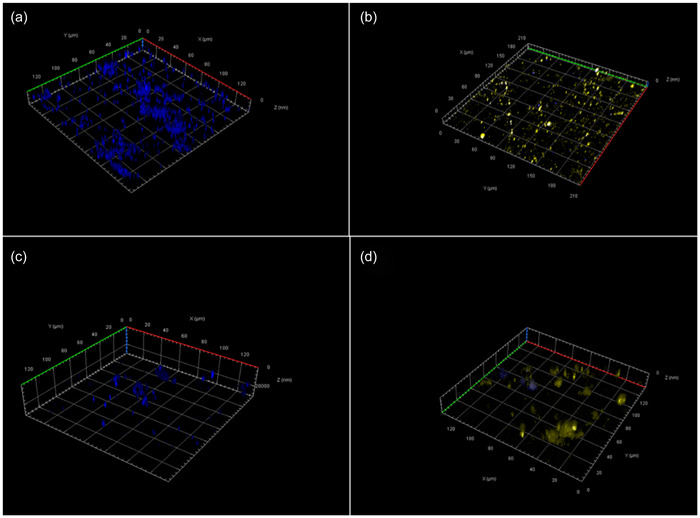
Confocal laser scanning microscopy comparison of ceramic surfaces inoculated with *Staphylococcus aureus* with no pretreatment (a) and inoculated following treatment with the *Bacillus* sp. blend (b), and nonpretreated surfaces challenged with *Escherichia coli* (c) and following treatment with the *Bacillus* sp. blend (d). *S. aureus* and *E. coli* are stained blue, with the *Bacillus* stained yellow.

**Figure A2:**
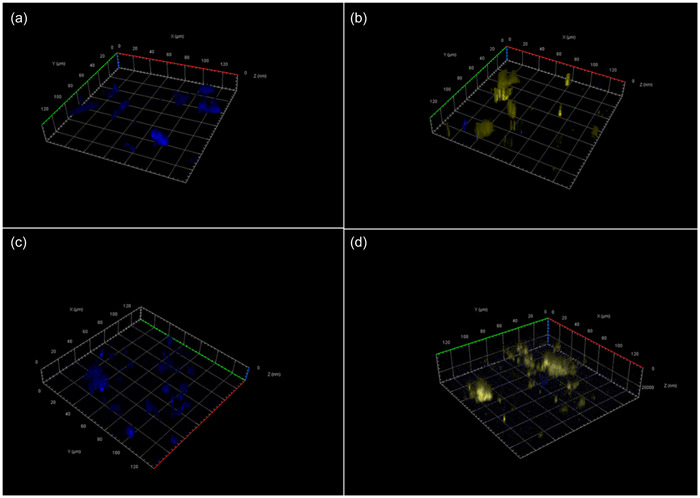
Confocal laser scanning microscopy comparison of melamine surfaces inoculated with *Staphylococcus aureus* with no pretreatment (a) and inoculated following treatment with the *Bacillus* sp. blend (b), and nonpretreated surfaces challenged with *Escherichia coli* (c) and following treatment with the *Bacillus* sp. blend (d). *S. aureus* and *E. coli* are stained blue, with the *Bacillus* stained yellow.

FISH‐CLSM imaging of the steel test surfaces indicated that upon coupons treated with PBS only, there was an attachment and proliferation of either *E. coli* or *S. aureus* that was not removed with washing (Figure 4). In contrast, when steel coupons were pretreated with the *Bacillus* blend, there was a reduction in the visible accumulation of either *E. coli* or *S. aureus*, with the majority of the surface instead showing fluorescence related to that of *Bacillus* spp. Similar observations were made on ceramic and melamine test pieces (Figures A1 and A2).

### 3.4 Antidry biofilm properties of the probiotic cleanser

**Figure 5:**
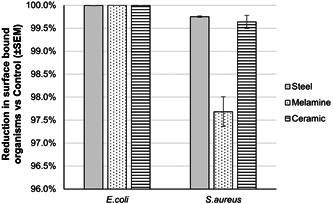
The probiotic cleanser (*Bacillus* spores and cleaning formulation) was capable of decreasing the microbial load on all three test surfaces when challenged with both *Escherichia coli* and *Staphylococcus aureus* compared to the phosphate‐buffered saline control.

When surfaces were pretreated with the probiotic cleanser formulation (*Bacillus* spores and cleaning formulation), there was no evidence of any recoverable organisms from steel, melamine, or ceramic when challenged with *E. coli*, representing a 100% reduction in dry biofilm compared to the control surfaces (Figure 5). There was a greater degree of variability observed across the *S. aureus*‐challenged test surfaces, where microbial loads were decreased by a minimum of 97.7% compared to the control surfaces. In the case of ceramic and steel surfaces, the reduction in viable biofilm‐associated cells was 99.6% ± 0.1% and 99.8% ± 0.0%, respectively.

## 4 DISCUSSION

There are clear challenges in producing methodologies for the testing of antimicrobial products against biofilms. While the testing of disinfectants within the BS:EN test methods mainly focus on surface tests such as BS EN 13697:2015 + A1:2019 (Anonymous, 2020) in which organisms are dried onto a surface over a short period (>1 h), those produced by ASTM have focused on the growth of biofilms using the CDC reactor (Biosurface Technologies, US [Anonymous, 2019]) but maintained in solution. In both cases, these test methods have a strong focus on the removal of biomass from a surface rather than the prevention of its accumulation. The data generated within this study suggest that with modifications, simple methodologies for the production of dry biofilms, such as those proposed by Adator et al. (2018), provide a simple but reproducible test model for assessing the ability of antimicrobials to prevent or reduce the formation of dry biofilms. The test method has also identified the potential for *Bacillus*‐based probiotics to act as a preventative measure to reduce the bioburden on surfaces associated with dry biofilms.

Through the use of the model system, it was demonstrated that *Bacillus* spp. spores contained within a probiotic cleanser were able to both germinate and persist upon a variety of hard surfaces. These observations tie in with those of Caselli et al. (2016, 2019), where similar levels and persistence were documented. The use of CLSM microscopy combined with FISH probes further reinforced microbial count data, showing the ability of *Bacillus* spp. to actively colonize steel, ceramic, and melamine surfaces. The ability of *Bacillus* spp. to actively colonize hard surfaces suggests that potential competitive exclusion mechanisms are at play between the added probiotic bacteria and pathogenic microorganisms. The use of three test surfaces (steel, ceramic, and melamine) demonstrated that the test can be easily modified to suit specific surface testing requirements. It is well documented that there are multiple properties concerning both the surfaces and microorganisms that could influence adhesion (Zheng et al., 2021), and variation in adhesion of both pathogen analogs and *Bacillus sp*. was also observed here. Further understanding of the metabolic activities of surface‐bound *Bacillus* spp. when exposed to clinically relevant pathogens would be of benefit to better understand the microbial mechanisms behind this. This data would be a valuable tool in refining bespoke consortia of different *Bacillus* species to enhance the efficacy of future probiotics products.

The impact of combining a hard surface cleaning formulation with a biological agent, in this case, *Bacillus* spp., was further understood in this work. The use of agents such as surfactants and chemical preservatives such as the common biocide BIT was able to provide an initial challenge to the pathogenic microorganisms but not the probiotic spores. This is due to the fact that the probiotic cleanser formulation was designed in a manner to provide a biocidal but not sporicidal challenge to preserve its integrity during storage. It is likely that as organic fouling and natural attrition diluted the chemical challenge, the concurrent germination of the probiotic spores allowed the *Bacillus* spp. to gain an immediate foothold in niche areas that could support microbial life.

## AUTHOR CONTRIBUTIONS

**Richard Wormald**: Data curation (equal); investigation (equal); methodology (equal); software (equal); visualization (equal); writing—review and editing (equal). **Paul N. Humphreys**: Conceptualization (equal); supervision (equal); writing—review and editing (equal). **Christopher J. Charles**: Conceptualization (equal); supervision (equal); writing—review and editing (equal). **Simon P. Rout**: Conceptualization (equal); funding acquisition (lead); methodology (equal); supervision (equal); visualization (equal); writing—original draft (lead).

## CONFLICT OF INTEREST STATEMENT

Christopher J. Charles is a current employee of Genesis Biosciences and was involved in the conceptualization of the project and manuscript review but had no role in the collection of data, analysis, or interpretation of the data. The remaining authors declare no conflict of interest.

## ETHICS STATEMENT

None required.

## Data Availability

All data are presented in this published article.

## 1

See Table A1 and Figures A1 and A2.

**Table A1:** Total recovery of *Bacillus* from surfaces.

	Log_10_ recovery	
Application	*Bacillus* only	*Bacillus* cleanser
Initial	6.82	6.92
Steel	6.78 ± 0.009	6.67 ± 0.09
Ceramic	6.55 ± 0.05	6.52 ± 0.03
Melamine	5.92 ± 0.2	6.02 ± 0.1
